# *Lactobacillus Rhamnosus* GG Affects the BDNF System in Brain Samples of Wistar Rats with Pepsin-Trypsin-Digested Gliadin (PTG)-Induced Enteropathy

**DOI:** 10.3390/nu12030629

**Published:** 2020-02-27

**Authors:** Antonella Orlando, Guglielmina Chimienti, Angela Maria Serena Lezza, Vito Pesce, Isabella Gigante, Benedetta D’Attoma, Francesco Russo

**Affiliations:** 1 Laboratory of Nutritional Pathophysiology, National Institute of Gastroenterology “S. de Bellis” Research Hospital, 70013 Castellana Grotte (Ba), Italy; antonella.orlando@irccsdebellis.it (A.O.); benedetta.dattoma@irccsdebellis.it (B.D.); 2 Department of Biosciences, Biotechnologies and Biopharmaceutics, University of Bari Aldo Moro, Via Orabona 4, 70100 Bari, Italy; guglielminaalessandra.chimienti@uniba.it (G.C.); angelamariaserena.lezza@uniba.it (A.M.S.L.); vito.pesce@uniba.it (V.P.); 3 Laboratory of Nutritional Biochemistry, National Institute of Gastroenterology “S. de Bellis” Research Hospital, 70013 Castellana Grotte (Ba), Italy; isabella.gigante87@gmail.com

**Keywords:** brain-derived neurotrophic factor, celiac disease, gliadin, Lactobacillus rhamnosus GG, probiotics, TrkB receptor

## Abstract

Celiac disease (CD) presents as chronic low-grade inflammation of the small intestine often characterized by psychiatric comorbidities. The brain-derived neurotrophic factor (BDNF), which we have shown to be reduced in the serum of CD patients, acts as the bridge between immune activation and the nervous system adaptive response. Since *Lactobacillus* has been shown to upregulate BDNF, this study aimed to evaluate whether the administration of *Lactobacillus rhamnosus* GG (L.GG) could positively affect the brain BDNF system in rats mimicking the CD lesions. Data have shown that the administration of pepsin-trypsin digested gliadin (PTG) and L.GG alter the levels of mature BDNF (mBDNF), as evaluated by Western blotting. PTG provoked a reduction of mBDNF compared to controls, and a compensatory increase of its receptor TrkB. L.GG induced a slight positive effect on mBDNF levels under normal conditions, while it was able to rescue the PTG-induced reduced expression of mBDNF. The curative effect of L.GG was finely tuned, accompanied by the reduction of TrkB, probably to avoid the effect of excessive BDNF.

## 1. Introduction

Celiac disease (CD) presents as a chronic low-grade inflammatory condition of the small intestine that develops in genetically susceptible individuals after the ingestion of gliadin [1]. Apart from classical gastrointestinal (GI) (such as diarrhea, bloating, abdominal pain, constipation) and extra-GI symptoms (e.g., iron deficiency anemia, weight loss, osteoporosis, and fatigue), it may often be characterized by psychiatric comorbidities [2], such as anxiety and depression [3]. Indeed, a link exists between inflammation and neuroplasticity: pro-inflammatory cytokines act as key mediators, with the brain-derived neurotrophic factor (BDNF) representing the molecular bridge between immune activation and the capacity of the nervous system to adapt to environmental challenges [4]. BDNF plays an important role in the development and regeneration of the central nervous system (CNS) [5]. It is also expressed in several peripheral tissues, including the GI tract [6], where it is released from enterocytes, enteric glial cells, and neurons. BDNF has been shown to affect peristalsis [7], the response of enteric neurons to pain-related neurotransmitters [8], as well as the modulation of intestinal barrier integrity [9]. 

The gene encoding for BDNF shows a quite complex structure: it contains eleven exons in humans [10] and nine exons in rodents [11], each having its promoter, allowing the production of multiple transcripts. All mRNAs encode the same protein since each transcript is constituted by a distinct 5’ untranslated exon spliced to the single 3’ coding exon. A sophisticated transcriptional regulation confers temporal and spatial control of BDNF expression in an activity-dependent manner. This regulation relies on distinct cellular signals that can activate the transcription at each of the 5’ promoters, such as the CREB-dependent upregulation of the promoter of exon IV by extracellular ATP [12], or the phorbol myristate acetate-driven induction of NF-kB (leading to activation of promoters of exons I, IV, and VI) [13]. Besides, the BDNF gene is also regulated during development by epigenetic modifications [14]. 

In addition to the transcriptional regulation, BDNF expression is also controlled at the post-transcriptional level. BDNF is initially synthesized as the proBDNF (32 kDa) that is subjected to a proteolytical cleavage to produce the mature protein (mBDNF, 14 kDa). According to the yin and yang theory of neurotrophins, the two isoforms exert different biological effects, having different receptors. Through its cognate tropomyosin related kinase receptor B (TrkB) and the non-specific neurotrophin receptor p75NTR, mBDNF promotes neuronal survival and enhances synaptic plasticity [15], while the proBDNF can exert an opposite effect by acting on p75NTR [16].

The clinical relevance of BDNF has been demonstrated in several inflammatory pathologies of the gut; the neurotrophin is downregulated in the brain of a murine model of inflammatory bowel disease [17], whereas it appeared to be upregulated in the intestinal mucosa [18], as well as in the serum [19] of patients suffering from diarrhea-predominant irritable bowel syndrome. Increased BDNF, related to inhibition of apoptotic processes, has been demonstrated in the inflamed enteric glia in the course of Crohn’s disease [20]. As concerns CD, data are conflicting: in a recent paper, our group reported decreased serum BDNF concentration in adult patients, both at the diagnosis and after a one-year gluten-free diet (GFD) [21], whereas Margoni et al. showed higher serum values of BDNF in pediatric CD patients than in healthy controls, which further increased after more than one year of GFD [22]. Since BDNF crosses the blood-brain barrier, it can be assumed that circulating levels of the protein reflects brain levels [23].

Currently, the introduction of a highly restrictive lifelong GFD is the gold standard treatment for CD. However, GFD is often associated with a reduced quality of life [21], and some patients are GFD nonresponsive [24]. Therefore, efforts are made to develop effective alternative treatments [25], such as the decreasing of gluten immunogenicity through genetically modified wheat [26], the use of HLA-DQ2 blockers, or vaccine therapy to prevent gluten-induced immune activation [27]. In consideration of the strong impact of gut microbiota on CD, increasing interest has developed on whether dysbiosis could be considered a risk factor for the disease [28] and on the effectiveness of probiotics/prebiotics as a supplementary therapy. Although the mechanisms by which probiotics/prebiotics could be beneficial for CD are yet to be clearly recognized, probiotics not only could provide the host with endopeptidases able to detoxify gluten [29] but also could affect host immune responses, thereby dampening the inflammatory burden [30]. Besides, the ability of probiotics to modulate BDNF functions through the modulation of gut microbiota has been reported [31].

Our group already demonstrated the protective effect on the intestinal barrier integrity of *Lactobacillus rhamnosus* GG (L.GG) in the course of pepsin-trypsin-digested gliadin (PTG)-induced enteropathy in new-born male Wistar rats [32]. In this framework, the present study aimed to investigate using the same experimental model, whether the administration of L.GG could positively affect the levels of BDNF. Since a bidirectional informational network between the gut and the brain has been determined [33], the possible response of the BDNF system to L.GG administration was investigated in brain samples of new-born male Wistar rats. Thus, the levels of pro and mBDNF isoforms, of different BDNF mature transcripts, and the TrkB and p75NTR receptors were evaluated.

## 2. Materials and Methods 

### 2.1. Gliadin Digest

PTG was obtained following a previously published procedure [34]. Briefly, 50 g wheat gliadin (Sigma-Aldrich, Milan, Italy) was dissolved in 500 mL 0.2 N HCl and digested for 2 h at 37 °C with 1 g pepsin (P6887, Sigma-Aldrich, Milan, Italy). After adjusting to pH 7.4, the peptic digest was incubated with 1 g trypsin (T7418, Sigma-Aldrich, Milan, Italy) for 4 h at 37 °C. After boiling for 30 min, the solution was freeze-dried, lyophilized, and stored at −20 °C until used.

### 2.2. Animals and Experimental Design

The study was approved by the Italian Ministry of Health (approval date: 15 December 2016; n. 1178/2016-PR). All the applied procedures followed the International Guidelines for the use of laboratory animals. Brain samples used in this study were from new-born Wistar rats housed at the animal housing room of the National Institute of Gastroenterology “S. De Bellis” Research Hospital, Castellana Grotte, Bari, Italy.

The experimental design included a control and four different treatments, and every litter of at least ten puppies represented a different treatment group (Table 1)

In detail, as concerns PTG treatment, new-born rats received orally 50 µg PTG/day in a single dose for ten days, and, finally, a provocative dose of PTG 100 µg two hours before sacrifice.

The probiotic *Lactobacillus rhamnosus* GG (ATCC 53103) (Dicoflor, Dicofarm, Rome, Italy) was administered orally at a concentration of 10^9^ CFU/day in a single dose for ten days.

After treatments, the puppies were sacrificed by anesthetic overdose, and brain samples were immediately removed and stored at −80 °C until assayed.

### 2.3. Western Immunoblotting

Proteins were extracted from brain samples using lysis buffer (Pierce Ripa buffer, Thermo Scientific, Rockford, IL, USA) supplemented with protease and phosphatase inhibitors (Thermo Scientific, Rockford, IL, USA).

From each sample, 30 µg of total proteins were denatured in 5 × Laemmli sample buffer and loaded into Any Kd pre-cast polyacrylamide gels (Bio-Rad, Milan, Italy) for Western blot analysis. Anti-BDNF 1:250 (MYBioSource, San Diego, CA, USA), anti-TrkB 1:1000, anti p75 NTR 1:500 (Thermo Scientific, Rockford, IL, USA) and β-actin 1:2000 (Santa Cruz Biotechnology, Santa Cruz, CA, USA) were used as primary antibodies. After overnight incubation, the membranes were further incubated with a horseradish peroxidase-conjugated rabbit secondary antibody (Bio-Rad, Milan, Italy). The proteins were detected by chemiluminescence (ECL, Thermo Scientific, Rockford, IL, USA) and immunoreactive bands were quantified using Image Lab Software (BioRad Laboratories Inc., Hercules, CA, USA) and normalized against β-actin expression.

### 2.4. PCR

In this study, the expression of BDNF total transcripts and transcripts containing exon III, IV and VI was evaluated.

Total cell RNA from brain samples was extracted using Tri-Reagent (Mol. Res. Center Inc., Cincinnati, OH, USA), following the manufacture’s instruction. For cDNA synthesis, about 2 μg total cell RNA was used. Reverse transcription (RT) was carried out using the iScript Advanced cDNA Synthesis Kit (BioRad Laboratories Inc., Hercules, CA, USA). 

Expression of BDNF total transcripts was evaluated by the quantitative PCR (qPCR) method. Real-time PCRs were performed in 25 µL final volume containing 2 µL of cDNA, 1x master mix with SYBR Green (iQ SYBR Green Supermix, BioRad Laboratories Inc., Hercules, CA, USA) and sense and antisense primers for the total BDNF and the GAPDH gene [35]. Reactions were performed in a CFX96 Real-Time PCR Detection System (Bio-Rad, Milan, Italy) using the following protocol: 45 cycles at 95 °C for 30 s, 95 °C for 5 s, 60 °C for 30 s followed by a melting curve step at 65–95 °C with a heating rate of 0.5 °C per cycle for 80 cycles. Relative quantification was done using the ΔΔCt method.

Expression of BDNF transcripts containing exon III, IV, and VI, respectively, were evaluated by end point-PCR using Dream Taq PCR 1x Master Mix (Thermo Fisher Scientific Inc, Waltham, MA, USA), 25 pmoles forward and reverse primers and cDNA template (2 µL) in 25 µL of final volume. PCR parameters were initial denaturation at 95 °C for 7 min, followed by 35 cycles of 30 s at 95 °C, 20 s at 60 °C, 30 s at 72 °C, and 7 min of final extension at 72 °C. Primers were as those in Smiljanic et al. [36].

An aliquot (10 µL) of each PCR amplification was loaded on 1.5% agarose gel. Ethidium bromide-stained bands were visualized using the Gel Doc XR documentation system (BioRad Laboratories Inc., Hercules, CA, USA) and Image Lab Software (BioRad Laboratories Inc., Hercules, CA, USA) was used to analyze band intensity. The expression of BDNF transcripts was expressed as relative values (OD of BDNF normalized to the OD of the corresponding GAPDH band).

### 2.5. Statistical Analysis

Due to the non-normal distribution of the data, non-parametric tests were performed. Data were analyzed by Kruskal–Wallis analysis of variance and Dunn’s Multiple Comparison Test. Correlation between pro and mBDNF levels was analyzed by the Spearman regression test. All data are expressed as mean and SEM. Differences were considered significant at *p* < 0.05. A specific software package (SigmaStat for Windows version 3.00 SPSS Inc. San Jose, CA, USA) was used.

## 3. Results

### 3.1. BDNF Analysis

**Figure 1:**
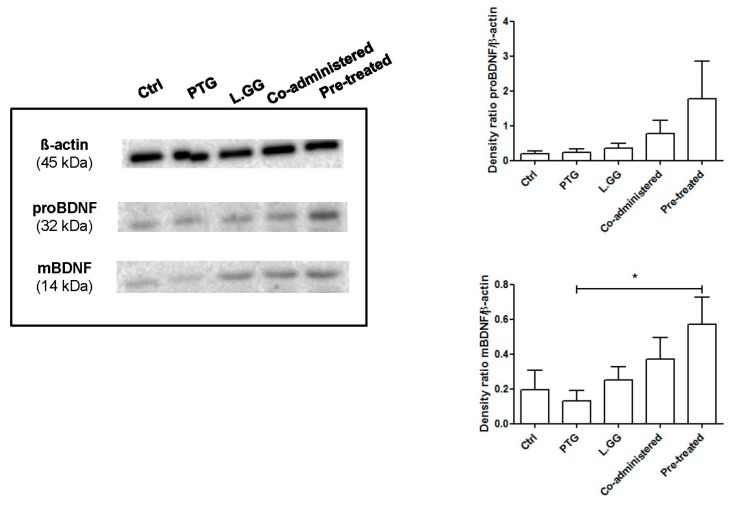
Western blot analysis of mBDNF and proBDNF in brain samples from control and treated rats, each group consisting of six rats. Immunoreactive bands were quantified using Image Lab Software (BioRad Laboratories Inc., Hercules, CA, USA). The graphs on the right show quantification of the intensity of bands calibrated to the intensity of the ß-actin band. All data represent the results of at least three independent experiments (mean plus SEM). * *p* < 0.05. Data were analyzed by Kruskal–Wallis analysis of variance and Dunn’s Multiple Comparison Test.

The levels of the mature BDNF and the BDNF precursor, reported as the expression relative to ß-actin, were evaluated in samples of the brain from untreated and treated rats. Western blot analyses showed the presence of both mBDNF and proBDNF isoforms (Figure 1). Experimental treatments affected the expression of the protein, with a statistically significant difference as concerns the mature isoform of BDNF (*p*: 0.0315, Kruskal–Wallis test). In rats sensitized with IFN-γ, PTG treatment induced a 33% reduction, and the administration of L.GG caused a small increase (29%) of mBDNF levels compared to Ctrl. When rats were co-administered with PTG and L.GG, a 91% increase in mBDNF levels compared to Ctrl occurred, whereas the administration of L.GG in pre-treated rats almost doubled the levels of mBDNF in comparison with Ctrl (194% increase); levels in co-treated rats were significantly different from those in PTG treated ones (*p* < 0.05, Dunn’s multiple comparison post-test) (Figure 1).

The precursor proBDNF was affected by the different treatments; the administration of PTG and L.GG, both alone and in combination, induced an increase in the levels of this isoform, although without reaching statistical significance (*p*: 0.234 Kruskal–Wallis test) (Figure 1).

**Figure 2:**
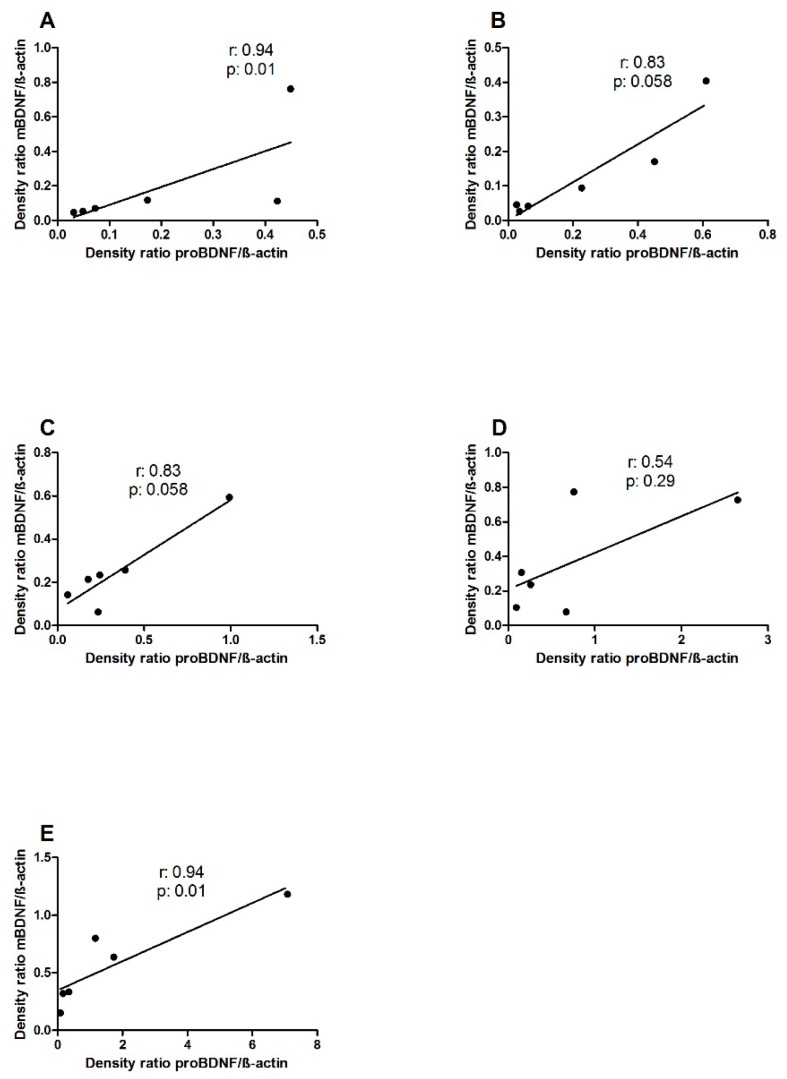
Correlation between proBDNF and mBDNF levels in brain samples from control and treated rats, each group consisting of six rats. **A**: Ctrl; **B**: PTG; **C**: L.GG; **D**: co-administered; **E**: pre-treated. Dots represent the values of at least three independent experiments. Data were analyzed by the Spearman Regression Test.

The levels of precursor and mature BDNF positively correlated, reaching statistical significance in the Ctrl and pre-treated groups (*r*: 0.94, *p*: 0.01, Spearman test) (Figure 2A,E, respectively) and approached significance in PTG and L.GG rats (*r*: 0.83, *p*: 0.058, Spearman test) (Figure 2B,C, respectively).

**Figure 3:**
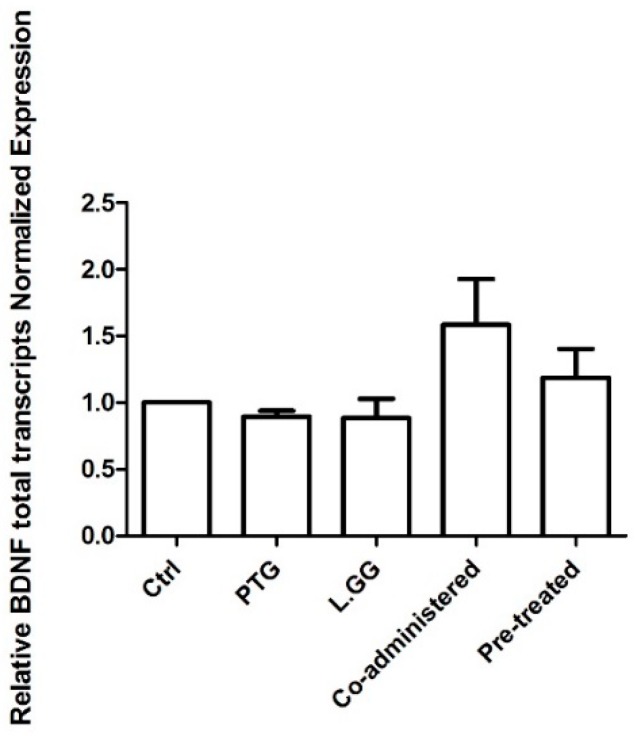
Quantitative PCR (qPCR) analysis of BDNF total transcripts in brain samples from control and treated rats, each group consisting of six rats. All data, represented as Fold Induction, are the results of at least three independent experiments (mean plus SEM). Data were analyzed by Kruskal–Wallis analysis of variance and Dunn’s Multiple Comparison Test.

The levels of BDNF total transcripts in brain samples from untreated and treated rats were determined. No statistically significant difference was found (Figure 3). 

**Figure 4:**
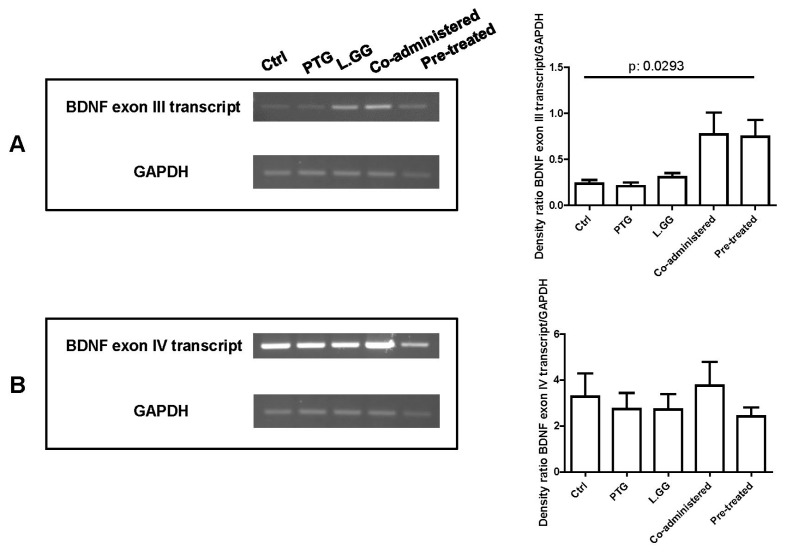
End point-PCR analysis of BDNF transcripts containing exon III (**A**) and IV (**B**) in brain samples from control and treated rats, each group consisting of six rats. An aliquot (10 µL) of each PCR amplification was loaded on 1.5% agarose gel. Bands were quantified using Image Lab Software (BioRad Laboratories Inc., Hercules, CA, USA). The graphs on the right show quantification of the intensity of bands calibrated to the intensity of the GAPDH band. All data represent the results of at least three independent experiments (mean plus SEM). Data were analyzed by Kruskal–Wallis analysis of variance and Dunn’s Multiple Comparison Test.

Figure 4 is a representative agarose gel showing end point-PCR results for exon III and IV respectively. Only faint bands were obtained using primers specific for the exon VI (data not shown). A statistically significant difference was found as concerns the transcript containing the exon III (*p*: 0.0293, Kruskal–Wallis test) (Figure 4A), but not for exon IV (Figure 4B).

### 3.2. TrkB and p75NTR Analysis 

**Figure 5:**
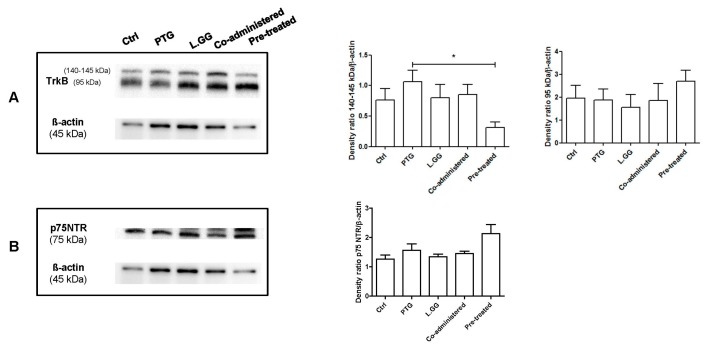
Western blot analysis of TrkB (**A**) and p75NTR (**B**) in brain samples from control and treated rats, each group consisting of six rats. Immunoreactive bands were quantified using Image Lab Software (BioRad Laboratories Inc., Hercules, CA, USA). The graphs on the right show quantification of the intensity of bands calibrated to the intensity of the ß-actin band. All data represent the results of at least three independent experiments (mean plus SEM). * *p* < 0.05. The data were analyzed by Kruskal–Wallis analysis of variance and Dunn’s Multiple Comparison Test.

The effect of PTG and L.GG administration on the levels of TrkB was evaluated. In brain samples, a 140–145 kDa and a 95 kDa TrkB isoforms were detected. The experimental treatments affected the levels of the 140–145 isoform (*p*: 0.0433, Kruskal–Wallis test). The administration of PTG induced a 39% increase in protein levels in comparison with Ctrl; an increase was also observed when L.GG was administered, both alone and in combination with PTG (5% and 12% increase, respectively). In contrast, the administration of L.GG after the PTG treatment induced a significant 59% reduction in protein levels compared to PTG (*p* < 0.05, Dunn’s multiple comparison post-test). Changes in the levels of the non-glycosylated form were not statistically significant (Figure 5A). 

Levels of the proBDNF p75NTR receptor were evaluated: experimental treatments did not significantly affect this protein (*p*: 0.0625, Kruskal–Wallis test) (Figure 5B).

## 4. Discussion

This study was performed to evaluate the effect of administration of the probiotic L.GG on the BDNF system in the course of PTG-induced enteropathy. An animal model able to mimic the CD lesions in vivo was used, namely, newborn rats sensitized with IFN-γ immediately after birth and administered with PTG [32,37].

In a previous study, our group reported not only lower circulating BDNF levels in CD patients than healthy controls, but also a significant correlation between low BDNF levels and impaired quality of life [21]. It has already been demonstrated in animal models that chronic GI inflammation induces anxiety-like behavior through the alteration of CNS biochemistry [38]. BDNF is a key molecule in the CNS, able to affect mood, behavior, learning, and memory and a reduced circulating level of this neurotrophin is related to the development of psychiatric disorders [39].

Since circulating BDNF levels reflect those in the brain [23], in the present study, the regulation of the BDNF system was evaluated in rat brain samples. Specifically, our aim was to investigate whether the administration of L.GG could modulate the expression of the mature, proteolytically cleaved mBDNF protein and the precursor proBDNF isoforms in the course of PTG induced enteropathy. Besides, total and differently spliced BDNF transcripts, the TrkB, evaluated as full-length and glycosylated forms, and p75NTR receptors levels were investigated. 

This study revealed that the experimental treatments (administration of PTG and L.GG, alone or in combination) significantly altered the levels of mBDNF, as evaluated by Western blotting. 

The administration of PTG to rats sensitized with IFN-γ downregulated the expression of the mature isoform of the neurotrophin, which is involved in promoting neuronal survival and plasticity [15]. This result is consistent with the already demonstrated relationship between peripheral inflammation and decreased brain neurotrophic factors [17], which can affect cognition, anxiety and depression [38,39,40], as well as with our previous findings in CD patients [21].

Increasing evidence shows that the gut microbiota can influence the development and maintenance of the CNS and enteric nervous system (ENS), through microbial metabolites, such as short-chain fatty acids and neurotransmitters, able to cross the intestinal and blood-brain barriers [41]. The effectiveness of probiotic supplementation in the reduction of psychological symptoms [42,43] and as an alternative treatment for CD [25] are nowadays the objects of investigation. Indeed, recent results from clinical trials [44], animal models [45], and in vitro studies [46] indicate that the *Lactobacillus* administration up-regulates BDNF expression.

The results of the present study show that the administration of L.GG was able to increase mBDNF compared to Ctrl rats slightly. When L.GG was co-administered with PTG, the increase in mBDNF was higher than that observed in rats treated with L.GG alone. This increase was even higher in PTG-pretreated rats, leading to significantly different neurotrophin levels from those in PTG rats. Therefore, L.GG showed only a slight positive effect on BDNF levels under normal conditions, while it was able to rescue the reduced expression of BDNF, thus repairing the adverse impact induced by a ten-day PTG administration.

The variations of proBDNF in all the experimental conditions followed a trend quite similar to that of the mature isoform, although the differences of proBDNF levels were not statistically significant. The existence of the balance between pro and mBDNF, shown by the positive correlation between the two isoforms, can be considered as suggestive of a major role of proBDNF as the precursor of the proteolytically cleaved mBDNF. Notably, an imbalance between the two isoforms and the up-regulation of the expression of proBDNF/p75NTR has been reported in patients with major depressive disorders [47].

The impact of experimental treatments on the levels of BDNF transcripts was also evaluated. It is noteworthy to remember the complexity of the regulation of BDNF expression, which involves transcriptional and post-transcriptional mechanisms, such as miRNA regulation [48]. Results from this study show the significant difference in the levels of the specific transcript containing exon III, one of the major activity-dependent transcripts [35]. Further studies are needed to dissect the complexity of the regulation of BDNF better.

mBDNF exerts its effect through the interaction with its specific TrkB receptor, which exists as multiple differently spliced isoforms, glycosylated and non-glycosylated [49]. The N-glycosylation, allowing the localization of the protein to the cell surface, enables ligand-specific activation [50]. Two forms of the TrkB protein were present in brain samples from differently treated rats, a 140–145 kDa one, representing the glycosylated receptor, and a 95 kDa form, corresponding to the full-length TrkB. The levels of the 95 kDa protein were not significantly affected by experimental treatments, whereas significant changes were observed in levels of the glycosylated protein. The most relevant increase in the 140–145 kDa protein was found in the PTG group, which could be indicative of a compensatory response to the reduction of its ligand mBDNF evoked by PTG treatment. Conversely, the reduction of the protein observed in the pre-treated group could be considered as protective against the exposure to possibly excessive mBDNF levels. Indeed, TrkB mediates neuronal necrosis in the case of elevated levels of mBDNF [51]. 

Consistently with the non-significant variation of pro-BDNF levels in all the experimental conditions, the levels of p75NTR also showed the same behavior.

In conclusion, data from this study demonstrate that the induced enteropathy affects the expression of the BDNF system in the rat brain. Interestingly, PTG treatment was able to provoke a balanced response consisting of a reduction of the neurotrophin compared to untreated controls along with a compensatory increase in the TrkB receptor. The curative effect of L.GG also appeared to be finely tuned in this experimental model, since the reduction of the cognate receptor accompanied the increase of BDNF in pre-treated rats to probably avoid the negative effect of an excess of BDNF [51].

On the basis of our results, the actual role of the BDNF system should be further investigated in gluten-related human disorders, as well as in the course of probiotic treatments. The obtained results could be helpful for the more efficient management of the celiac patient.

## Figures and Tables

**Table 1:** Experimental groups.

	Group	Treatment	Age (Days)
**1**	Ctrl	Animals not treated	10
**2**	PTG	Animals previously sensitized with 1000 U IFN-γ administered intraperitoneally after birth and with a following oral administration of PTG 50 µg/day for 10 days	10
**3**	L.GG	Animals treated with an oral administration of L.GG 1 × 10^9^ CFU for 10 days	10
**4**	Co-administered	Animals previously sensitized with 1000 U IFN-γ administered intraperitoneally after birth and with a following oral co-administration of PTG 50 µg/day and L.GG 10^9^ CFU for 10 days	10
**5**	Pre-treated	Animals previously sensitized with 1000 U IFN-γ administered intraperitoneally after birth with a following administration of PTG for 10 days and successively treated with L.GG 10^9^ CFU for further 10 days	20

Ctrl = controls. PTG = pepsin-trypsin-digested gliadin. L.GG = *Lactobacillus rhamnosus* GG.

## References

[1] Balakireva A.V., Zamyatnin A.A. (2016). Properties of Gluten Intolerance: Gluten Structure, Evolution, Pathogenicity and Detoxification Capabilities. Nutrients.

[2] Slim M., Rico-Villademoros F., Calandre E.P. (2018). Psychiatric Comorbidity in Children and Adults with Gluten-Related Disorders: A Narrative Review. Nutrients.

[3] Campagna G., Pesce M., Tatangelo R., Rizzuto A., La Fratta I., Grilli A. (2017). The progression of coeliac disease: Its neurological and psychiatric implications. Nutr. Res. Rev..

[4] Calabrese F., Rossetti AC., Racagni G., Gass P., Riva M.A., Molteni R. (2014). Brain-derived neurotrophic factor: A bridge between inflammation and neuroplasticity. Front. Cell. Neurosci..

[5] Arévalo J.C., Wu SH. (2006). Neurotrophin signaling: Many exciting surprises!. Cell. Mol. Life Sci..

[6] Lommatzsch M., Braun A., Mannsfeldt A., Botchkarev V.A., Botchkareva N.V., Paus R., Fischer A., Lewin G.R., Renz H. (1999). Abundant production of brain-derived neurotrophic factor by adult visceral epithelia. Implications for paracrine and target-derived Neurotrophic functions. Am. J. Pathol..

[7] Grider J.R., Piland B.E., Gulick M.A., Qiao L.Y. (2006). Brain-derived neurotrophic factor augments peristalsis by augmenting 5-HT and calcitonin gene-related peptide release. Gastroenterology.

[8] Boesmans W., Gomes P., Janssens J., Tack J., Vanden Berghe P. (2008). Brain-derived neurotrophic factor amplifies neurotransmitter responses and promotes synaptic communication in the enteric nervous system. Gut.

[9] Yu Y.B., Zhao D.Y., Qi Q.Q., Long X., Li X., Chen F.X., Zuo XL. (2017). BDNF modulates intestinal barrier integrity through regulating the expression of tight junction proteins. Neurogastroenterol. Motil..

[10] Pruunsild P., Kazantseva A., Aid T., Palm K., Timmusk T. (2007). Dissecting the human BDNF locus: Bidirectional transcription, complex splicing, and multiple promoters. Genomics.

[11] Aid T., Kazantseva A., Piirsoo M., Palm K., Timmusk T. (2007). Mouse and rat BDNF gene structure and expression revisited. J. Neurosci. Res..

[12] Takasaki I., Takarada S., Tatsumi S., Azegami A., Yasuda M., Fukuchi M., Tabuchi A., Kondo T., Tabuchi Y., Tsuda M. (2008). Extracellular adenosine 5’-triphosphate elicits the expression of brain-derived neurotrophic factor exon IV mRNA in rat astrocytes. Glia.

[13] Morioka N., Yoshida Y., Nakamura Y., Hidaka N., Hisaoka-Nakashima K., Nakata Y. (2013). The regulation of exon-specific brain-derived neurotrophic factor mRNA expression by protein kinase C in rat cultured dorsal root ganglion neurons. Brain Res..

[14] Chen K.W., Chen L. (2017). Epigenetic Regulation of BDNF Gene during Development and Diseases. Int. J. Mol. Sci..

[15] Dechant G., Barde Y.A. (2002). The neurotrophin receptor p75 (NTR): Novel functions and implications for diseases of the nervous system. Nat. Neurosci..

[16] Lu B., Pang P.T., Woo NH. (2005). The yin and yang of neurotrophin action. Nat. Rev. Neurosci..

[17] Do J., Woo J. (2018). From Gut to Brain: Alteration in Inflammation Markers in the Brain of Dextran Sodium Sulfate-induced Colitis Model Mice. Clin. Psychopharmacol. Neurosci..

[18] Zhang Y., Qin G., Liu D.R., Wang Y., Yao S.K. (2019). Increased expression of brain-derived neurotrophic factor is correlated with visceral hypersensitivity in patients with diarrhea-predominant irritable bowel syndrome. World J. Gastroenterol..

[19] Russo F., Chimienti G., Riezzo G., Linsalata M., D’Attoma B., Clemente C., Orlando A. (2018). Adipose Tissue-Derived Biomarkers of Intestinal Barrier Functions for the Characterization of Diarrhoea-Predominant IBS. Dis. Mark..

[20] Steinkamp M., Schulte N., Spaniol U., Pflüger C., Hartmann C., Kirsch J., von Boyen G.B. (2012). Brain derived neurotrophic factor inhibits apoptosis in enteric glia during gut inflammation. Med. Sci. Monit..

[21] Russo F., Chimienti G., Clemente C., Ferreri C., Orlando A., Riezzo G. (2017). A possible role for ghrelin, leptin, brain-derived neurotrophic factor and docosahexaenoic acid in reducing the quality of life of coeliac disease patients following a gluten-free diet. Eur. J. Nutr..

[22] Margoni D., Michalakakou K., Angeli E., Pervanidou P., Kanaka-Gantenbein C., Chrousos G., Papassotiriou I., Roma E. (2018). Serum brain-derived neurotrophic factor in children with coeliac disease. Eur. J. Clin. Invest..

[23] Lommatzsch M., Zingler D., Schuhbaeck K., Schloetcke K., Zingler C., Schuff-Werner P., Virchow J.C. (2005). The impact of age, weight and gender on BDNF levels in human platelets and plasma. Neurobiol. Aging..

[24] Cichewicz A.B., Mearns E.S., Taylor A., Boulanger T., Gerber M., Leffler D.A., Drahos J., Sanders D.S., Thomas Craig K.J., Lebwohl B. (2019). Diagnosis and Treatment Patterns in Celiac Disease. Dig. Dis. Sci..

[25] Serena G., Kelly C.P., Fasano A. (2019). Nondietary Therapies for Celiac Disease. Gastroenterol. Clin. North Am..

[26] van den Broeck H.C., van Herpen T.W., Schuit C., Salentijn E.M., Dekking L., Bosch D., Hamer R.J., Smulders M.J., Gilissen L.J., van der Meer I.M. (2009). Removing celiac disease-related gluten proteins from bread wheat while retaining technological properties: A study with Chinese Spring deletion lines. BMC Plant. Biol..

[27] Yoosuf S., Makharia G.K. (2019). Evolving Therapy for Celiac Disease. Front. Pediatr..

[28] Girbovan A., Sur G., Samasca G., Lupan I. (2017). Dysbiosis a risk factor for celiac disease. Med. Microbiol. Immunol..

[29] Di Cagno R., De Angelis M., Auricchio S., Greco L., Clarke C., De Vincenzi M., Giovannini C., D’Archivio M., Landolfo F., Parrilli G. (2004). Sourdough bread made from wheat and nontoxic flours and started with selected lactobacilli is tolerated in celiac sprue patients. Appl. Environ. Microbiol..

[30] Suez J., Zmora N., Segal E., Elinav E. (2019). The pros, cons, and many unknowns of probiotics. Nat. Med..

[31] Maqsood R., Stone T.W. (2016). The Gut-Brain Axis, BDNF, NMDA and CNS Disorders. Neurochem. Res..

[32] Orlando A., Linsalata M., Bianco G., Notarnicola M., D’Attoma B., Scavo M.P., Tafaro A., Russo F. (2018). Lactobacillus rhamnosus GG Protects the Epithelial Barrier of Wistar Rats from the Pepsin-Trypsin-Digested Gliadin (PTG)-Induced Enteropathy. Nutrients.

[33] Wang H.X., Wang Y.P. (2016). Gut Microbiota-brain Axis. Chin. Med. J. (Engl.).

[34] Drago S., El Asmar R., Di Pierro M., Grazia Clemente M., Tripathi A., Sapone A., Thakar M., Iacono G., Carroccio A., D’Agate C. (2006). Gliadin, zonulin and gut permeability: Effects on celiac and non celiac intestinal mucosa and intestinal cell lines. Scand. J. Gastroenterol..

[35] Liang S., Wang T., Hu X., Luo J., Li W., Wu X., Duan Y., Jin F. (2015). Administration of Lactobacillus helveticus NS8 improves behavioral, cognitive, and biochemical aberrations caused by chronic restraint stress. Neuroscience.

[36] Smiljanic K., Pesic V., Mladenovic Djordjevic A., Pavkovic Z., Brkic M., Ruzdijic S., Kanazir S. (2015). Long-term dietary restriction differentially affects the expression of BDNF and its receptors in the cortex and hippocampus of middle-aged and aged male rats. Biogerontology.

[37] Laparra J.M., Olivares M., Gallina O., Sanz Y. (2012). Bifidobacterium longum cect 7347 modulates immune responses in a gliadin-induced enteropathy animal model. PLoS ONE.

[38] Bercik P., Verdu E.F., Foster J.A., Macri J., Potter M., Huang X., Malinowski P., Jackson W., Blennerhassett P., Neufeld K.A. (2010). Chronic gastrointestinal inflammation induces anxiety-like behavior and alters central nervous system biochemistry in mice. Gastroenterology.

[39] Autry A.E., Monteggia L.M. (2012). Brain-derived neurotrophic factor and neuropsychiatric disorders. Pharmacol. Rev..

[40] Simon K.U., Neto E.W., Tramontin N.D.S., Canteiro P.B., Pereira B.D., Zaccaron R.P., Silveira P.C.L., Muller A.P. (2019). Intranasal insulin treatment modulates the neurotropic, inflammatory, and oxidant mechanisms in the cortex and hippocampus in a low-grade inflammation model. Peptides.

[41] Heiss C.N., Olofsson L.E. (2019). The role of the gut microbiota in development, function and disorders of the central nervous system and the enteric nervous system. J. Neuroendocrinol..

[42] Pirbaglou M., Katz J., de Souza R.J., Stearns J.C., Motamed M., Ritvo P. (2016). Probiotic supplementation can positively affect anxiety and depressive symptoms: A systematic review of randomized controlled trials. Nutr. Res..

[43] Nikolova V., Zaidi S.Y., Young A.H., Cleare A.J., Stone J.M. (2019). Gut feeling: Randomized controlled trials of probiotics for the treatment of clinical depression: Systematic review and meta-analysis. Ther. Adv. Psychopharmacol..

[44] Hwang Y.H., Park S., Paik J.W., Chae S.W., Kim D.H., Jeong D.G., Ha E., Kim M., Hong G., Park S.H. (2019). Efficacy and Safety of Lactobacillus Plantarum C29-Fermented Soybean (DW2009) in Individuals with Mild Cognitive Impairment: A 12-Week, Multi-Center, Randomized, Double-Blind, Placebo-Controlled Clinical Trial. Nutrients.

[45] Choi J., Kim Y.K., Han P.L. (2019). Extracellular Vesicles Derived from Lactobacillus plantarum Increase BDNF Expression in Cultured Hippocampal Neurons and Produce Antidepressant-like Effects in Mice. Exp. Neurobiol..

[46] Cheng R., Xu T., Zhang Y., Wang F., Zhao L., Jiang Y., He F. (2019). Lactobacillus rhamnosus GG and Bifidobacterium bifidum TMC3115 Can Affect Development of Hippocampal Neurons Cultured In Vitro in a Strain-Dependent Manner. Probiotics Antimicrob. Proteins..

[47] Zhou L., Xiong J., Lim Y., Ruan Y., Huang C., Zhu Y., Zhong J.H., Xiao Z., Zhou X.F. (2013). Upregulation of blood proBDNF and its receptors in major depression. J. Affect. Disord..

[48] Karpova N.N. (2014). Role of BDNF epigenetics in activity-dependent neuronal plasticity. Neuropharmacology.

[49] Otani K., Okada M., Yamawaki H. (2017). Diverse distribution of tyrosine receptor kinase B isoforms in rat multiple tissues. J. Vet. Med. Sci..

[50] Watson F.L., Porcionatto M.A., Bhattacharyya A., Stiles C.D., Segal R.A. (1999). TrkA glycosylation regulates receptor localization and activity. J. Neurobiol..

[51] Kim H.J., Hwang J.J., Behrens M.M., Snider B.J., Choi D.W., Koh J.Y. (2003). TrkB mediates BDNF-induced potentiation of neuronal necrosis in cortical culture. Neurobiol. Dis..

